# Genome Sequence of *Serratia marcescens* Phage BF

**DOI:** 10.1128/genomeA.00211-17

**Published:** 2017-06-08

**Authors:** Eoghan Casey, Brian Fitzgerald, Jennifer Mahony, Gabriele Andrea Lugli, Marco Ventura, Douwe van Sinderen

**Affiliations:** a School of Microbiology, University College Cork, Cork, Ireland; b APC Microbiome Institute, University College Cork, Cork, Ireland; c Laboratory of Probiogenomics, Department of Chemistry, Life Sciences and Environmental Sustainability, University of Parma, Parma, Italy

## Abstract

Phages infecting *Serratia marcescens*, a common causative agent of nosocomial infections, have potential therapeutic applications. Here, we report the complete genome of the novel *S. marcescens* phage BF, representing the third-largest phage genome sequenced to date.

## GENOME ANNOUNCEMENT

*Serratia marcescens* is a Gram-negative, rod-shaped bacterium belonging to the family *Enterobacteriaceae* (1). Isolation of phages infecting this species may be applied to control nosocomial infections of *S. marcescens* (2). To date, the genomes of just two phages, myovirus η (NC_021563) and PS2 (NC_024121; unknown morphology), infecting *S. marcescens* are available from the NCBI viral genome database (https://www.ncbi.nlm.nih.gov/genomes).

Environmental sample screening led to the isolation of *S. marcescens* UCC2017, verified by 16S rRNA sequencing, from composted grass. Five grams of fresh compost were diluted in 50 mL of one-quarter-strength Ringer’s solution and filtered through a 0.45-μm membrane. This filtrate was then assessed for phages infecting *S. marcescens* UCC2017 using the double-layer spot assay method (3), in which a zone of clearing indicates phage lysis. Despite repeated attempts, the isolated phage (hereafter named BF) was unable to produce plaques. Therefore, BF propagation was achieved by picking the zone of lysis and incubating this with 50 mL of *S. marcescens* UCC2017 culture for 6 h. Following filtration (0.45 µm) and DNase treatment of the lysate, bacteriophage DNA was isolated employing a phage DNA isolation kit (Norgen Biotek, Canada).

Illumina MiSeq sequencing (mean coverage depth of 21.55-fold) was performed, followed by genome assembly using MIRA version 4.0.2 through the MEGAnnotator pipeline (4). Quality of the final contig was improved using the Burrows-Wheeler aligner, the SAMtools suite, and VarScan version 2.2.3. Coding DNA sequence (CDS) predictions were performed with Prodigal version 2.06. Annotation of predicted CDSs was performed using BLAST against sequences present in the NCBI database and HMMER against the Pfam database (http://pfam.xfam.org). Transfer RNA gene prediction was performed with tRNAscan-SE version 1.21.

The BF genome measures 357,154 bp, with a G+C content of 35.3% and 549 predicted CDSs. This makes it the third-largest bacteriophage genome sequenced to date, following *Bacillus megaterium* phage G (498 kb) and *Cronobacter* sp. phage GAP32 (358 kb); this allows for the classification of BF as a “jumbo” phage (5). Comparative genome analysis revealed the closest relatives of BF to be *Cronobacter* sp. phage GAP 32 (6) (436 protein homologues, E-value <0.001) and *Pectobacterium* sp. phage CBB (7) (445 protein homologues, E-value <0.001), both “jumbo” members of the family *Myoviridae*. Both CBB and GAP32 belong to the Rak2-like group (named after Rak2, a *Klebsiella*-infecting phage [8]). Based on the extensive homology between BF and these Rak2-like members, we propose to assign BF to this phage group.

The novelty of the BF genome allows functional predictions for 165 of the 549 deduced gene products with no evident modular genome organization. HHPred analysis predicted small clusters, such as the phage baseplate (BF_0232 to BF_0241), which encodes two potential receptor-binding proteins (RBPs)—the product of BF_0258, showing structural similarity to the coliphage T5 RBP, and that of BF_0259, which is similar to the carbohydrate-binding domains of *C. perfringens* (9)—suggesting that the BF receptor is saccharidic in nature. BF also encodes five putative chromosome condensation proteins (BF_0546 to BF_0550), similar to that specified by just a single copy in GAP32 (6) and presumably aiding in packaging the large genome into the capsid.

### Accession number(s).

The BF genome has been deposited in the NCBI/GenBank database under the accession number KY630187.
